# Magnetic, Electrical, and Mechanical Behavior of Fe-Al-MWCNT and Fe-Co-Al-MWCNT Magnetic Hybrid Nanocomposites Fabricated by Spark Plasma Sintering

**DOI:** 10.3390/nano10030436

**Published:** 2020-02-29

**Authors:** Alexandre Tugirumubano, Sun Ho Go, Hee Jae Shin, Lee Ku Kwac, Hong Gun Kim

**Affiliations:** 1 Institute of Carbon Technology, Jeonju University, Jeonju-si 55069, Korea; alexat123@yahoo.com (A.T.); royal2588@naver.com (S.H.G.); ostrich@jj.ac.kr (H.J.S.); kwac29@jj.ac.kr (L.K.K.); 2 Department of Mechanical and Automotive Engineering, Jeonju University, Jeonju-si 55069, Korea

**Keywords:** magnetic hybrid nanocomposites, nanoparticles, magnetic properties, mechanical properties, spark plasma sintering

## Abstract

This paper aims to investigate different properties of the Fe-Al matrix reinforced with multi-walled carbon nanotube (MWCNT) nanocomposites with the Al volume content up to 65%, according to the Fe-Al combination. In addition, the effect of adding Co content on the improvement of the soft magnetic properties of the nanocomposites was carried out. The nanocomposites were fabricated using the powder metallurgy process. The iron-aluminum metal matrix reinforced multi-walled carbon nanotube (Fe-Al-MWCNT) nanocomposites showed a continuous increase of saturation magnetization from 90.70 A·m^2^/kg to 167.22 A·m^2^/kg and microhardness, whereas the electrical resistivity dropped as the Al content increased. The incorporation of Co nanoparticles in Fe-Al-MWCNT up to 35 vol% of Co considerably improved the soft magnetic properties of the nanocomposites by reducing the coercivity and retentivity up to 42% and 47%, respectively. The results showed that Al-based magnetic nanocomposites with a high Al volume content can be tailored using ferromagnetic particles. The composites with a volume content of magnetic particles (Fe+Co) greater than 60 vol% exhibited higher saturation magnetization, higher coercivity, and higher retentivity than the standard Sendust core. Moreover, the produced composites can be used for the lightweight magnetic core in electromagnetic devices due to their low density and good magnetic and mechanical properties.

## 1. Introduction

Recently, the requirements for lightweight and high performance in various engineering structures and technologies have tremendously attracted many researchers and industries to composite materials. Among these composites, aluminum metal matrix composites are interesting and promising materials to fulfill the need for lightweight structure because of their excellent strength-to-weight and stiffness-to-weight ratio [1,2,3]. The final properties of these composites are strongly influenced by the reinforcing materials and the fabrication process. The mechanical, electrical, and thermal properties and corrosion behavior of the Al-based composites have been studied by many investigators, and improvement of these properties was achieved depending on the reinforcement and process parameters [4,5,6]. However, currently, there are no significant studies on the improvements in magnetic properties of those composites. Furthermore, very few studies can be found on the Fe-Al magnetic materials with high Al content.

The use of iron oxide (*α*-Fe_2_O_3_) nanoparticles as reinforcement in aluminum matrix composites were reported to produce Al-based magnetic composites [7,8,9]. However, these composites were found to present high coercivity and low saturation magnetization due to the poor magnetic properties of iron oxide [7]. The aluminum metal matrix composites reinforced with 30% of iron oxide Fe_3_O_4_ have shown a saturation magnetization of 13.43 A·m^2^/kg, and the Fe_3_O_4_ reinforcement was found to reduce the electrical conductivity of the composites [10,11]. Fathy et al. [12] investigated the magnetic properties of Al metal matrix reinforced with a 5% to 15% content of Fe particles as magnetic reinforcement. Although they found an enhancement of magnetic properties, the saturation magnetization of their composites was substantially low (less than 1 A·m^2^/kg). This was attributed to the formation of a diamagnetic Al_13_Fe_4_ intermetallic compound during the manufacturing process. These magnetic properties seem to be relatively low for most magnetic applications such as the magnetic core in transformers, inductors, or other electromagnetic devices. Therefore, in order to have metal matrix magnetic composites with lightweight and good magnetic properties, new approaches are advised.

In this paper, Al particles and Fe nanoparticles were combined to form a dual matrix and reinforced with 2 vol% of multi-walled carbon nanotubes (MWCNTs). It is well known that aluminum is a lightweight material, compared to iron, and is paramagnetic. On the other hand, iron is a heavy metal and is a ferromagnetic material with the best saturation induction compared to other ferromagnetic materials. The multi-walled carbon nanotubes (MWCNTs) are known to be advanced, light, and strong materials which have different applications, including the strengthening of materials in various composite materials [13,14,15]. The MWCNTs have good electric, mechanical, and thermal properties, and they are promising as reinforcements in composite materials. In this work, the Fe-Al-MWCNT hybrid nanocomposites were produced by spark plasma sintering with the aluminum content varying between 28 and 65 vol%. Their morphology and magnetic, electrical, and mechanical properties were tested and evaluated according to the Fe and Al volume-percentage combination. In addition, the effect of Fe and Co combination on various properties was studied by maintaining the Al and MWCNT contents in composites constant.

## 2. Materials and Methods

### 2.1. Sample Preparation

The powders of iron (Fe) nanoparticles with an average particle size of 90–110 nm and purity of 99.9%, aluminum (Al) powders with an average particle size of 30–40 μm and purity of 99.9%, and multi-walled carbon nanotubes (MWCNTs) with a 8–15 nm diameter and a 5–20 µm length were prepared with a volume ratio based on a sample of 50 mm in diameter and 30 mm in length. Furthermore, the cobalt (Co) nanoparticles with an average particle size of 80–120 nm and purity of 99.8% were prepared and used to evaluate the effect of Co on the magnetic properties of the composites with the above-mentioned materials. The Fe, Al, and Co powders were supplied by Ditto Technology Co. Ltd., Gunpo, Korea, whereas MWCNT powders were supplied by Nanosolution, Jeonju, Korea. Table 1 and Table 2 show the prepared samples according to the volume fraction of iron, aluminum, and cobalt nanoparticle powders, respectively.

The powders were mixed using the ball-milling technique on a horizontal roll ball mill. Before milling, 2 g of stearic acid were added as the process agent to reduce the cold welding of the powder and limit the powders sticking to the walls of the ball mill jar. Sixty alumina balls with a 10-mm diameter were used as the milling media. The powder milling was performed with a milling speed of 300 rpm for 48 h [9].

In order to prepare the powders for sintering, graphite die and two punches were prepared. Graphite sheets were used as die-wall and punch lubricant. The powder mixtures were filled in graphite die and then initially compacted with a hydraulic press to adjust and equalize the outer length of the punches (upper punch and bottom punch). The die containing the pre-compacted powders was mounted in a spark plasma sintering (SPS) machine (SPS-3.20MK-V, SPS Syntex Inc., Kawasaki, Japan) for simultaneous compaction and sintering. This process was performed by applying an initial pressure of 20 MPa while increasing the temperature from room temperature to 500°C. From 500°C to 600°C, a pressure of 40 MPa was applied, maintained for 10 min at 600°C, and then completely released during SPS-chamber cooling from 600°C to room temperature. The obtained cylindrical samples were machined into specimens using an electrical discharge machine (EDM) for characterization.

### 2.2. Characterization

Scanning electron microscopy (SEM, CX-200, COXEM Co.Ltd., Daejeon, Korea) and field-emission scanning electron microscopy (FE-SEM, JSM-7100F, Jeol, Tokyo, Japan), combined with an energy dispersive X-ray spectrometer (EDS; Aztec Energy, Oxford Instruments, Abingdon, UK), were used to investigate the morphology and elemental distribution of the composites. X-ray powder diffraction (XRD, CuK*α*: *λ* = 1.54 Å) was used to examine the crystallography and phase identification.

The cubic specimens of 2 mm × 2 mm × 2 mm were prepared and tested for magnetic property measurements using a vibrating sample magnetometer (VSM 7404-S, Lakeshore Cryotronics, Inc., Westerville, OH, USA) with an applied field varying form –1 T to +1 T. The bar sample preparation and the transverse-rupture strength (TRS) testing were performed according to the ASTM standard B528 [16], using a universal tensile-testing machine. The TRS testing was conducted by considering three specimens for each sample. The Vickers microhardness was also tested with an applied load of 0.1 kgf for 10 s using a hardness tester (HM-123, Code No 810-990K, Mitutoyo Corporation, Kawasaki, Japan). For each sample, 10 measurements were taken and averaged for microhardness characterization. The electrical resistivity was measured using the four-probe method on the rectangular samples having the same size as the TRS specimens (i.e., 31.7 mm in length, 12.7 mm in width, and 6.35 in thickness). For each material, 20 measurements were taken and averaged.

## 3. Results and Discussion

### 3.1. Microstructure Analysis of the As-Received Powders and Fe-Al-MWCNT Nanocomposites

**Figure 1:**
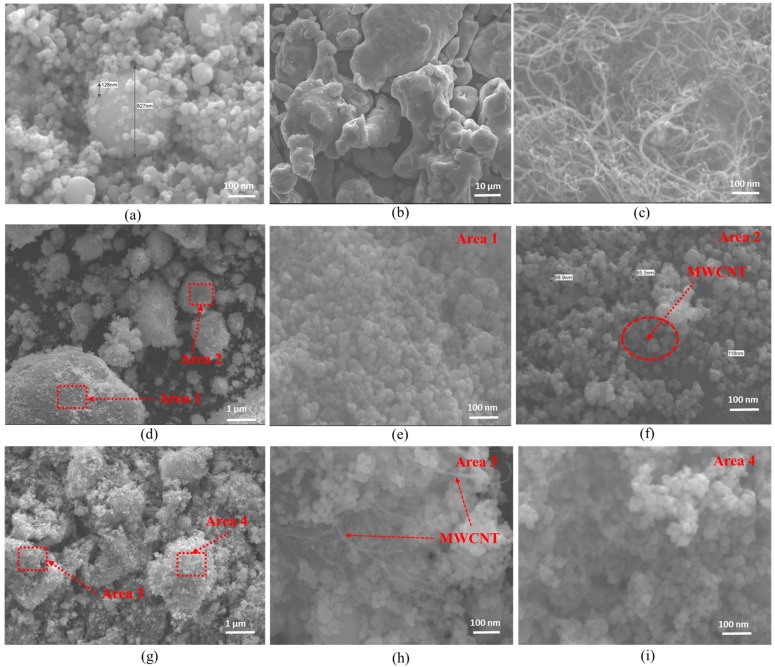
SEM images of powders: (**a**) As-received Fe nanopowders; (**b**) As-received Al powders; (**c**) As-received MWCNT; (**d**) Ball-milled (38Fe-60Al-2MWCNT) powders; (**e**) and (**f**) Higher Magnification of Selected Areas (1&2) in the images (**d**); (**g**) Ball-milled (65Fe-33Al-2MWCNT) powders; (**h**) and (**i**) Higher Magnification of Selected Areas (3&4) in the images (**g**).

Figure 1 shows the SEM images of the starting materials. The starting Fe powders shown in Figure 1a revealed a mixture of very small Fe particles (at nanoscale) and relatively big Fe particles. The iron (Fe) particles were of spherical shape. The Al particles shown in Figure 1b seemed to be large even at low magnification because they were at microscale. Figure 1c shows that the MWCNT were in the form of fibers with an entangled network. Figure 1d–i shows that the core shells of nanoparticles were created on the surface of the large particles after ball milling. It can be seen that a larger amount of nanoparticles were bonded on the big particles when the nanocomposites had a higher volume content of Fe nanoparticles (i.e., 65Fe-33Al-2MWCNT; Figure 1g–i) than when they had a lower volume content of Fe nanoparticles (i.e., 38Fe-60Al-2MWCNT; Figure 1d–f). The dispersion of MWNCT could also be observed at a high magnification of the ball-milled powders, as seen in Figure 1f,h.

**Figure 2:**
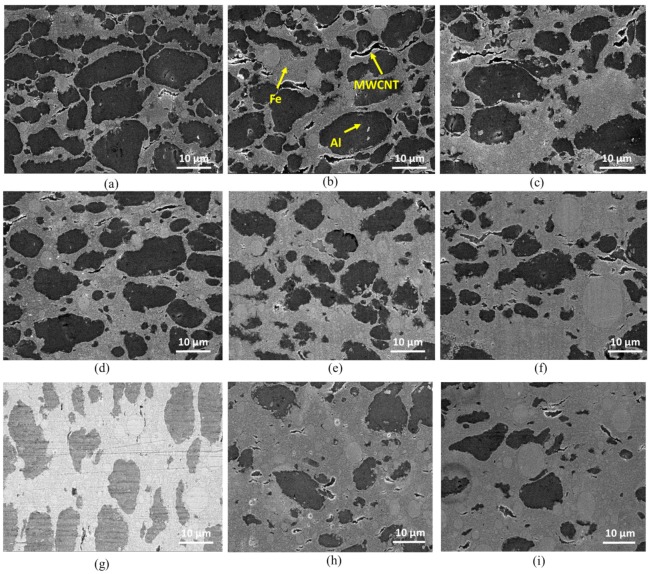
SEM images of Fe-Al-MWCNT hybrid nanocomposites: (**a**) 33Fe-65Al-2MWCNT (M1); (**b**) 38Fe-60Al-2MWCNT (M2); (**c**) 43Fe-55Al-2MWCNT (M3); (**d**) 48Fe-50Al-2MWCNT (M4); (**e**) 50Fe-48Al-2MWCNT (M5); (**f**) 55Fe-43Al-2MWCNT (M6); (**g**) 60Fe-38Al-2MWCNT (M7); (**h**) 65Fe-33Al-2MWCNT (M8); and (**i**) 70Fe-28Al-2MWCNT (M9).

Figure 2 shows SEM images of the composites. The Fe, Al, and MWCNTs are presented in the light gray, dark gray, and black colors, respectively, and are clearly indicated in Figure 2b. It is clear that Fe, Al, and MWCNTs were well distributed in the composites and the higher the volume content of Fe was, the larger its surface area was. The SEM images show that at a lower Fe content the Fe nanoparticles formed thin layers around the Al particles, and those layers got thicker as the volume fraction of Fe nanoparticles increased. The layers of Fe nanoparticles were the results of nanoparticles coating on the surface of large particles, using the mechanical ball-milling technique as discussed in previous research [17,18].

At a Fe volume content greater than 50% (Figure 2e–i), the Al particles were distributed in Fe phases as reinforcing particles in the Fe matrix. It can be seen that most of MWCNTs were trapped inside the Fe areas. This was because a large number of iron nanoparticles had surrounded the Al particles and the clusters of MWCNTs as a result of ball milling. The SEM images of the composites revealed the presence of big spherical Fe particles in the Fe area. However, it can be seen that the total surface formed by the big spherical Fe particles seemed smaller than the rest of the Fe area in the SEM images. Therefore, it can be believed that the volume content of big Fe particles was smaller than the volume content of small particles (at nanoscale) which may explain the average particle size of 90–110 nm (as specified by the iron powder supplier Ditto Technology Co. Ltd.).

**Figure 3:**
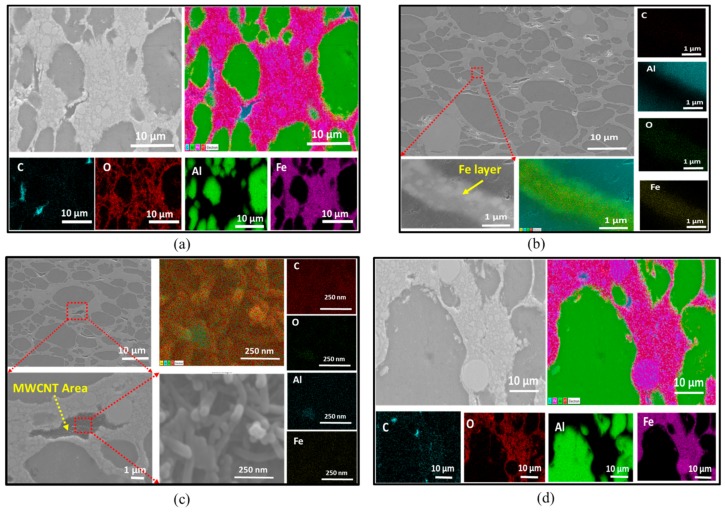
Energy Dispersive Spectroscopy (EDS) mapping: (**a**) 33Fe-65Al-2MWCNT (M1); (**b**) Higher magnification of selected region in 33Fe-65Al-2MWCNT (M1); (**c**) Higher magnification of selected region in 38Fe-60Al-2MWCNT (M2) with MWCNT area; and (**d**) 65Fe-33Al-2MWCNT (M8).

The elemental mappings performed using EDS are shown in Figure 3. The elements present in the materials can distinctively be seen in the areas of each element illustrated by their own colors. Figure 3a,d shows the distribution of elements in a composite with lower Fe volume content (33Fe-65Al-2MWCNT) and in a composite with higher Fe volume content (65Fe-33Al-2MWCNT), respectively. The EDS images show the presence of oxygen (O) which covers the surface of the iron (Fe) area. However, it can be seen that a high concentration of oxygen (O) is rather lower on the surface area of the large Fe particles than the surface area occupied by small Fe particles. This suggests that the area formed by small Fe particles were more oxidized than the area formed by big Fe particles. One possible reason for the presence of oxygen (O) in the composites may be the contamination of the sample during the ball milling due to the use of alumina balls as the ball-milling media. The second reason may be the oxidation of the surface of the samples after being polished for SEM and EDS analyses as the samples were exposed to the air. Figure 3b displays the selected area with a layer of Fe nanoparticles between Al particles and its EDS mapping at the high magnification of ×50,000 in the 33Fe-65Al-2MWCNT nanocomposite. Figure 3d shows the SEM image of 38Fe-60Al-2MWCNT nanocomposites, the high magnification of the selected area in the nanocomposites, and the MWCNT area that was mapped with the magnification of ×150,000. The image clearly shows the entangled MWCNTs.

**Figure 4:**
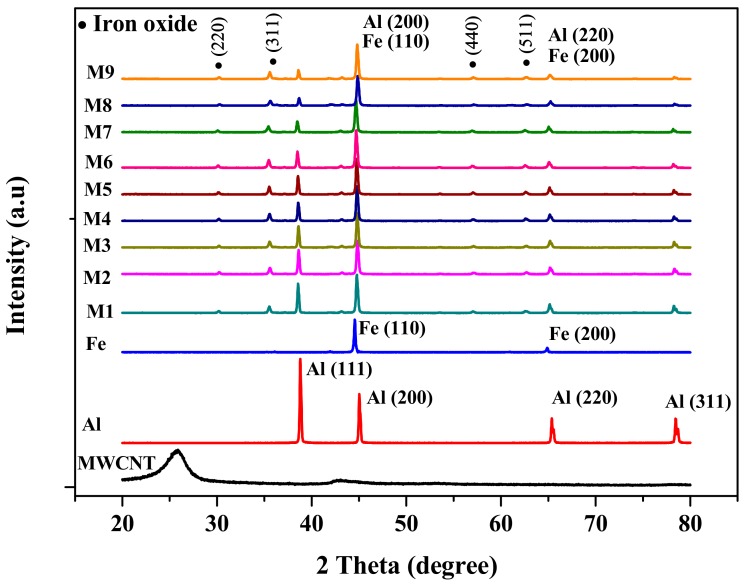
X-ray diffraction (XRD) patterns of Fe-Al-MWCNT hybrid nanocomposites.

The X-ray diffraction (XRD) analysis was conducted on the polished surface of the composites to investigate the formation of phases in Fe-Al-MWCNT nanocomposites. The XRD patterns of the composites in comparison to that of MWCNTs, Al, and Fe are shown in Figure 4. The Al XRD peaks (111), (200), (220), and (311), and the Fe XRD peaks (110) and (200) were detected in the XRD patterns of the composites. The intensity of Al peaks (111) and (311) decreased as the Al content decreased from 65 vol% (M1) to 28 vol% (M9). It can be seen that the iron oxide peaks (220), (311), (440), and (511) appeared in the XRD patterns of the composites which revealed the formation of iron oxide. This might have resulted from either the exposition to the air of polished surface before testing or the oxygen contamination during the ball-milling process. The XRD patterns of the composites did not show peaks of the MWCNTs or carbide compound which suggests that no carbide formation may have occurred as it did in some previous studies on carbon-nanotube-reinforced aluminum composites [19,20,21].

### 3.2. Magnetic Properties of Fe-Al-MWCNT Hybrid Nanocomposites

**Figure 5:**
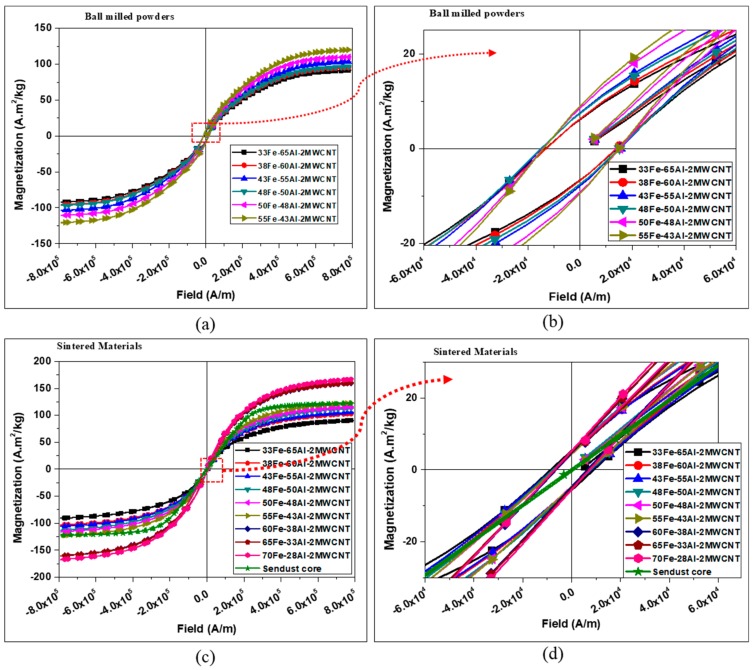
Magnetic hysteresis loops of the nanocomposites: (**a**) Full loops of ball-milled powder; (**b**) Magnification of selected region in (**a**); (**c**) Full loops of sintered composites; and (**d**) Magnification of selected region in (**c**).

**Figure 6:**
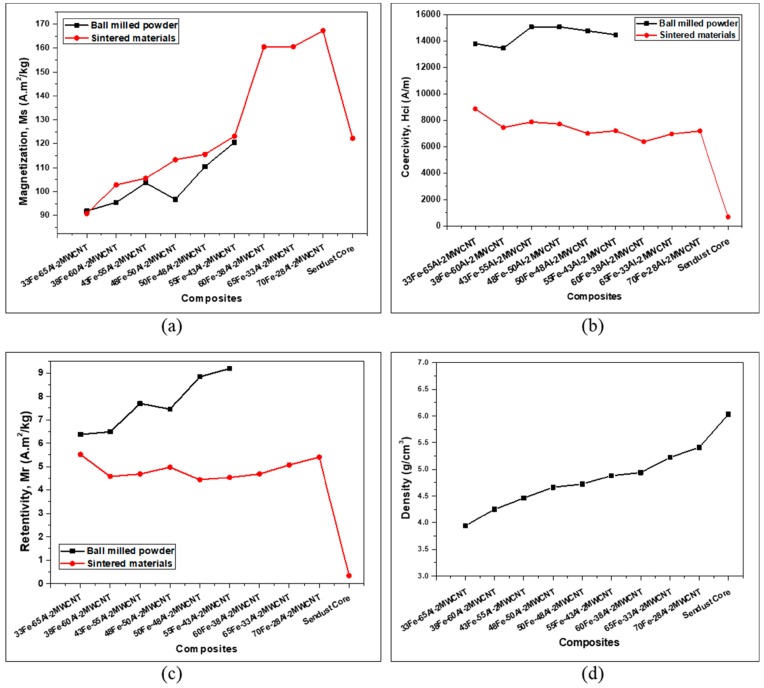
Magnetic properties and density of Fe-Al-MWCNT nanocomposites: (**a**) Saturation magnetization; (**b**) Coercivity; (**c**) Retentivity; and (**d**) Volume density.

The saturation magnetization, coercivity, and retentivity of the composites were recorded by VSM. Figure 5a,b and Figure 5c,d show the hysteresis loops of chosen powders after ball milling and the sintered composites, respectively. For comparison, the conventional Sendust core (CS610125, Fe-Si-Al alloy powder manufactured by Chang Sung Corporation, Korea) was purchased and tested with VSM measurements. The results of the magnetic properties of Sendust core were compared with the manufactured composites as shown in Figure 5c,d and Figure 6a,c. The Fe-Al-MWCNT composites had the hysteresis loops similar to that of standard Sendust core which is a ferromagnetic material. Thus, the Fe-Al-MWCNT nanocomposites can be considered ferromagnetic materials. It can be seen that the ball-milled powders and compacts exhibited ferromagnetic behavior.

Figure 6 compares the specific saturation magnetization, intrinsic coercivity, and retentivity of the powders and sintered composites. It was found that the saturation magnetizations of the compacts were, in general, higher than that of the ball-milled powders, and they increased as the content of Fe nanoparticles was increased. On the other hand, the coercivity and retentivity were comparatively reduced after sintering. A decrease of 35% to 51% in coercivity and 13% to 51% in retentivity was achieved as a result of the sintering of composites with Fe volume content varying from 33 vol% to 55 vol%. In fact, the coercivity and retentivity were high for the as-milled powders because of the presence of large gaps between particles when they were packed for measurement. However, the compact and sintering played an essential role in improving the densification of the nanocomposites by eliminating those gaps and therefore reducing the coercivity and retentivity and enhancing the saturation magnetization. Moreover, the stearic acid used as a process agent during ball milling stayed in its solid form after ball milling. But stearic acid was gradually decomposed while increasing the sintering temperature, leading to a lower volume of non-magnetic materials which contributed to the reduction of magnetic dilution and coercivity. Therefore, it can be inferred that it would be difficult to predict the trend of magnetic properties (especially coercivity and retentivity) based on the mixture of powders before the compaction and sintering process.

The reduction of coercivity and retentivity narrowed the hysteresis loops and consequently reduced the hysteresis losses of materials because the hysteresis losses of magnetic material are proportional to the area of the hysteresis loop. For the compacted composites, the composite with the lowest Fe content (33 vol%) had the highest coercivity of 8875 A/m, whereas the coercivity of the other composites fluctuated between 15085.99 A/m and 6383.20 A/m (see Figure 6b). The saturation magnetization, coercivity, and retentivity of the Sendust core obtained by VSM measurements were 122.20 A·m^2^/kg, 685.10 A/m, and 0.33 A·m^2^/kg, respectively. Evidently, the Fe-Al-MWCNT composites with Fe content of 55 vol% and above exhibit greater saturation magnetization (123–167 A·m^2^/kg) than the commercial Sendust core, but their coercivity and retentivity are extremely higher than that of the Sendust core (see Figure 5 and Figure 6). Figure 5g shows that the Fe-Al-MWCNT composites have wider hysteresis loops than the conventional Sendust core, and Figure 6b shows that the Fe-Al-MWCNT composites have a coercivity greater than 5000 A/m. Therefore, since the Fe-Al-MWCNTs exhibit the coercivity in a range between 1000 and 100,000 A/m, they can be classified as semi-hard magnetic materials [22].

As shown in Figure 6d, a continuous increase in density from 3.94 g/cm^3^ at low Fe content to 5.41 g/cm^3^ at high Fe content was observed which was attributed to the high volume content of iron phase with high density (7.87 g/cm^3^).

### 3.3. Electrical and Mechanical Properties of Fe-Al-MWCNT Hybrid Nanocomposites

**Figure 7:**
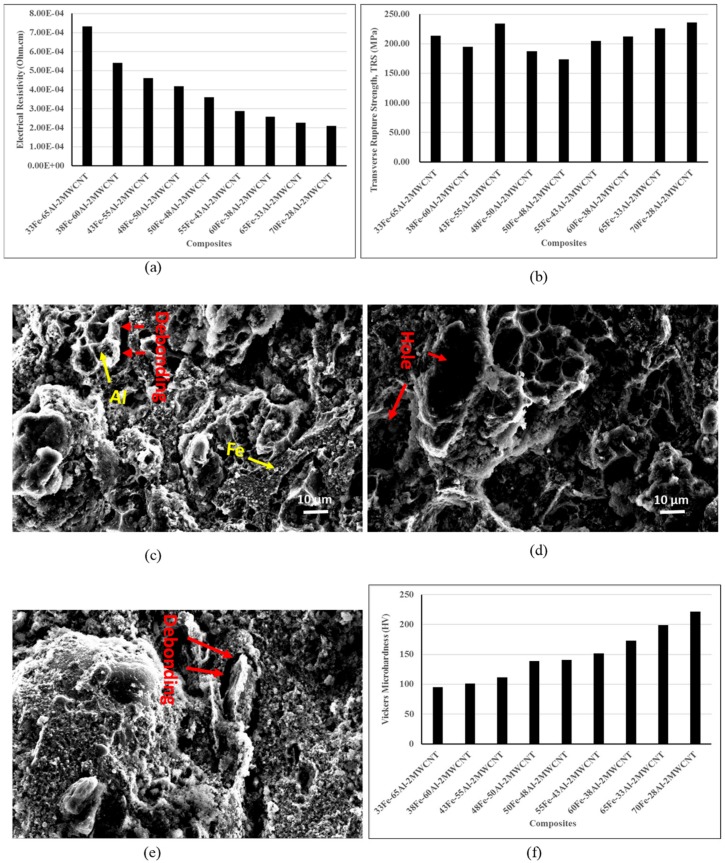
Electrical and mechanical properties of Fe-Al-MWCNT composites: (**a**) Electrical resistivity, (**b**) Mean transverse rupture strength (TRS); (**c**) Fractography SEM image of 33Fe-65Al-2MWCNT; (**d**) Fractography SEM image of 50Fe-48Al-2MWCNT; (**e**) Fractography SEM image of 70Fe-28Al-2MWCNT; and (**f**) Microhardness.

The electrical resistivity of Fe-Al-MWCNT nanocomposites was measured using the four-probe method. The results analyzed in Figure 7a are the average resistivity of 20 recorded data for each sample. The uninterrupted drop in electrical resistivity was observed as the iron content was increased while reducing the Al content. The decrease in electrical resistivity of Fe-Al-MWCNT composites due to the reduction of Al content was in agreement with the variation electrical properties of Fe-Al based alloys [23].

The transverse rupture strength of the Fe-Al-MWNCT nanocomposites was evaluated by using the three-point bending test method. The three-point bending method of evaluating the transverse rupture strength consisted of applying a load to the center of the bar specimen. Then the load at which the sample broke was used to calculate the transverse rupture strength (TRS; MPa), using the following Equation (1) [16]:

(1) $$TRS=\frac{3PL}{2t^{2}w}$$

where *P* is the maximum load at rupture (*N*), *L* is the length specimen span relative to the fixture (25.4 mm, according to ASTM B528), *t* and *w* are the thickness and width of the specimens, respectively.

**Figure 8:**
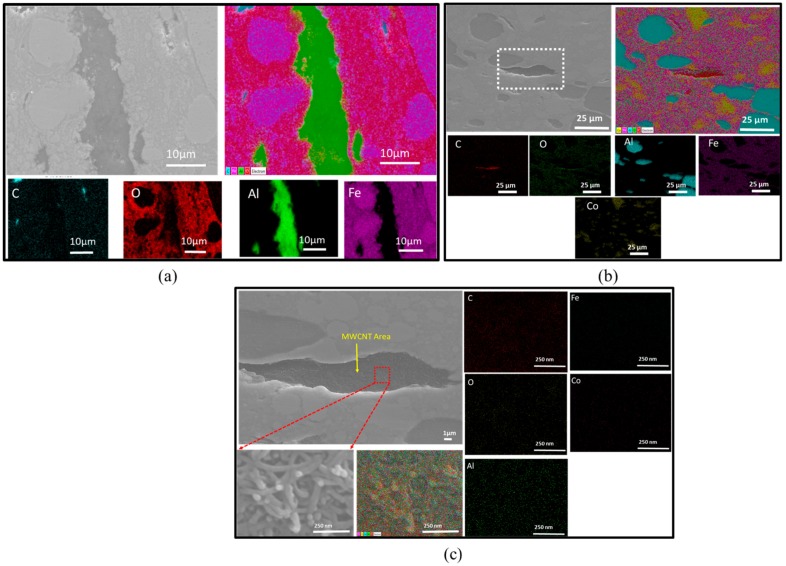
EDS mapping of the hybrid nanocomposites: (**a**) 70Fe-28Al-2MWCNT; (**b**) 60Fe-10Co-28Al-2MWCNT; and (**c**) High magnification of a selected region in (**b**) for 60Fe-10Co-28Al-2MWCNT.

Figure 8b shows the average transverse rupture strength of three specimens tested for each material. The results showed that the lowest strength of 173.83 MPa was given by the 50Fe-48Al-2MWCNT (M5), whereas the highest strength of 234.39 MPa was given by 43Fe-55Al-2MWCNT (M3). It can be seen in Figure 7b that for the composites with Fe content lower than 50 vol%, the strengths were decreased as the Al content decreased, although this trend was interrupted by a sudden increase of strength for composites 43Fe-55Al-2MWCNT (M3). However, the composites with a Fe content greater than 50 vol%, the transverse rupture strength continuously increased up to a maximum of 235.99 MPa with an increase in iron volume content. In fact, at a high-volume content of aluminum, it was seen from SEM images (Figure 2a–d) that Fe nanoparticles formed brittle coating layers around ductile Al particles. Therefore, at this level, the transverse rupture strength of the composites was mainly handled by the elasticity and plasticity of aluminum particles. As the volume ratio of iron nanoparticles increased, the Fe layer thickness increased which led to an expansion of space between Al particles and an increased brittleness of the composites due to insufficient bonding between ductile Al particles, MWCNTs, and brittle Fe nanoparticles. Consequently, a decrease in strength was observed. However, when the Al content was decreased from 48 vol% to 28 vol% while increasing Fe content from 50 vol% to 70 vol%, it was seen in the SEM images (Figure 2e–i) that large areas of Fe phases were formed, and the Al particles were randomly distributed in the Fe matrix. It can be assessed for the nanocomposites with high Fe content that the resistance to the applied load and load transfer in materials were mainly ensured by strong bonding of Fe nanoparticles that formed the iron matrix, leading to the enhancement of mechanical strength as the Fe content was augmented. The surface fracture analysis of the selected composites 33Fe-65Al-2MWCNT, 50Fe-48Al-2MWCNT, and 70Fe-28Al-2MWCNT was carried out after the transverse rupture strength testing, as shown in Figure 7c–e. It can be seen in fractography images that the Fe domains underwent brittle failure, whereas the failure of Al domains was ductile. The fracture of nanocomposites was mainly due to the combined debonding of Al to Fe and brittleness of Fe areas. The debonding between Al particles and Fe domain created holes on the fracture surface as can be seen in Figure 7d.

Figure 7f shows the microhardness obtained by averaging 10 measurements for each specimen. The results showed a continuous enhancement of hardness as the content of iron nanoparticles augmented. The lowest hardness was HV94.92 and the highest was HV 221.49 for the higher Al content and lower Al content, respectively. Thus, an increase of 57.14% in hardness was achieved by augmenting the Fe content from 33 vol% to 70 vol% and reducing the Al content. This can evidently be attributed to the higher hardness of iron grains than the Al grains. The improvement of microhardness as a function of Fe content in Fe-Al based composites was in good agreement with the results reported by Fathy et al. [12].

### 3.4. Effect of Co Content on the Properties of (70-x)Fe-xCo-28Al-2MWCNT Hybrid Nanocomposites (x = 0 to 35 vol%)

#### 3.4.1. Morphology

Figure 8a,b shows the EDS mapping of the selected composites with high Fe content (70Fe-28Al-2MWCNT and 60Fe-10Co-28Al-2MWCNT). Figure 8 distinctively shows the area of each element in the composites. The EDS mapping displayed oxygen (O) over the surface of iron. Similar to the previous samples (Fe-Al-MWCNT composites), in Figure 3, a higher concentration of oxygen (O) was detected on the surface occupied by small Fe particles than the area occupied by large Fe particles. This indicated the possible oxidation of iron that might have occurred after polishing the samples and being exposed to the air before being visualized with EDS. There may have also been contamination during the preparation of powder mixtures before sintering as a result of the exposure of powders to the air while weighing and the residues of alumina balls that were left during the ball milling. Figure 8c shows an EDS analysis image of a multi-walled carbon nanotube (MWCNT) area map at a magnification of ×150 000.

#### 3.4.2. Magnetic and Electrical Properties

**Figure 9:**
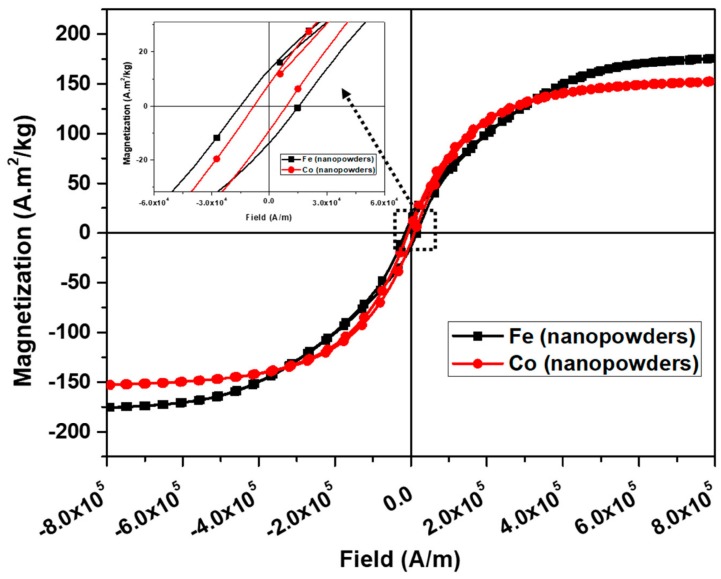
Hysteresis curves of as-received Fe and Co nanoparticles.

In order to improve the soft magnetic properties of Fe-Al-MWCNT nanocomposites, the Co nanoparticles were incorporated in the Fe-Al-MWCNT composite with best magnetic properties. The hysteresis curves of the as-received Fe and Co nanoparticles that were obtained using VSM are plotted in Figure 9. The iron nanoparticles had relative higher coercivity and higher saturation magnetization and consequently wider hysteresis loop than cobalt. The Fe and Co nanoparticles had the saturation magnetization of 175.79 A·m^2^/kg and 152.91 A·m^2^/kg, respectively. Their coercivities were 15309.71 A/m and 8425.96 A/m, respectively.

**Figure 10:**
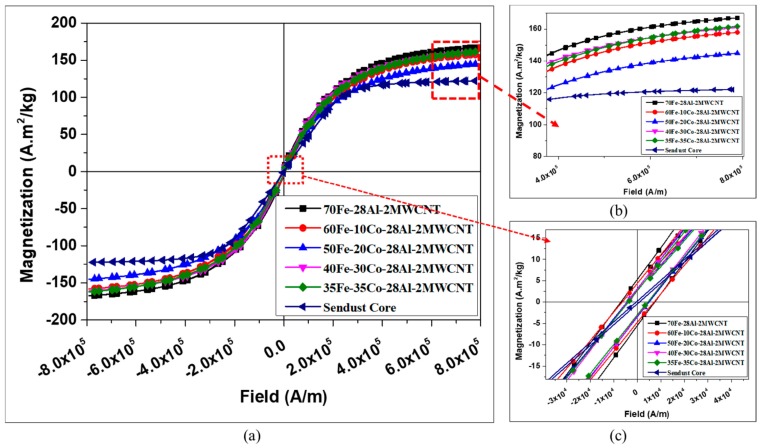
Comparison of hysteresis loops of the Fe-Al-MWCNT and Fe-Co-Al-MWCNT composites: (**a**) Full loops; (**b**) and (**c**) magnification of selected regions.

**Figure 11:**
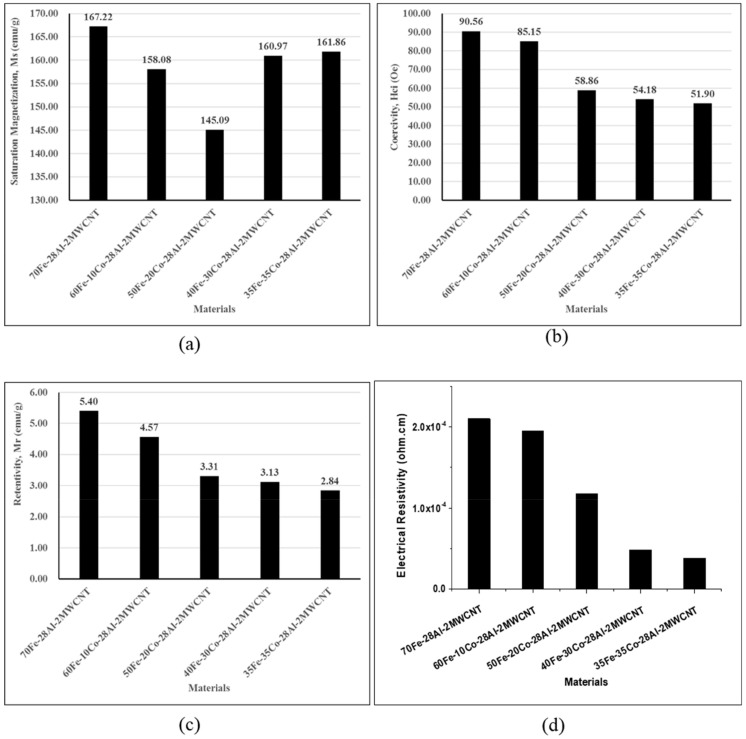
Magnetic and electrical properties of the Fe-Co-Al-MWCNT composites: (**a**) Saturation magnetization; (**b**) Coercivity; (**d**) Retentivity; and (**c**) Electrical resistivity.

Figure 10 shows the hysteresis curves of Fe-Co-Al-MWCNT nanocomposites, according to the Fe-Co combination in comparison with the hysteresis curve of commercial Sendust core (CS610125, Fe-Si-Al alloy powder). The typical magnetic properties of the composites extracted from the hysteresis curves are plotted in Figure 11. The saturation magnetization, shown in Figure 11a, were found to decrease using Co content up to 20 vol% and then start to increase when the Co volume fraction was increased. This might be caused by the modification of magnetic ordering in the composite and crystallinity of materials as the composition ratio changed. However, all Fe-Co based nanocomposites had lower saturation magnetization than the 70Fe-28Al-2MWCNT composite (without Co, 167.22 A·m^2^/kg). In Fe-Co based nanocomposites, 50Fe-20Co-28Al-2MWCNT exhibited the lowest saturation magnetization of 145.09 A·m^2^/kg, whereas 35Fe-35Co-28Al-2MWCNT exhibited the highest saturation magnetization of 161.86 A·m^2^/kg.

The coercivity (Figure 11b) and retentivity (Figure 11c) dropped by increasing the volume content of Co nanoparticles. The coercivity reduced from 7210.19 A/m to 4132.01 A/m when the volume content of Co increased from 0 vol% to 35 vol%. This shows a 42.7% drop in coercivity. The retentivity also decreased due to high Co content, and it continuously declined to 47.4% when 35 vol% of Co was used, in comparison to the 70Fe-28Al-2MWCNT nanocomposite. The composites exhibited higher saturation magnetization, higher coercivity, and higher retentivity than the standard Sendust core.

The electrical resistivity obtained from four-probe measurements for each composite is shown in Figure 11d. It is clear, in Figure 11d, that the use of cobalt nanoparticles led to a drop of electrical resistivity, and consequently, to an enhancement of electrical conductivity. The value of resistivity for the 70Fe-28Al-2MWCNT nanocomposite was 2.10 × 10^−4^ Ω.cm which continuously decreased as the Co content increased when the Fe-Co combination was used in composites. The lowest resistivity of 3.82 × 10^−5^ Ω.cm was given by the 35Fe-35Co-28Al-2MWCNT composite. This may be associated with lower resistivity of Co when compared to that of Fe.

#### 3.4.3. Mechanical Properties and Volume Density

**Figure 12:**
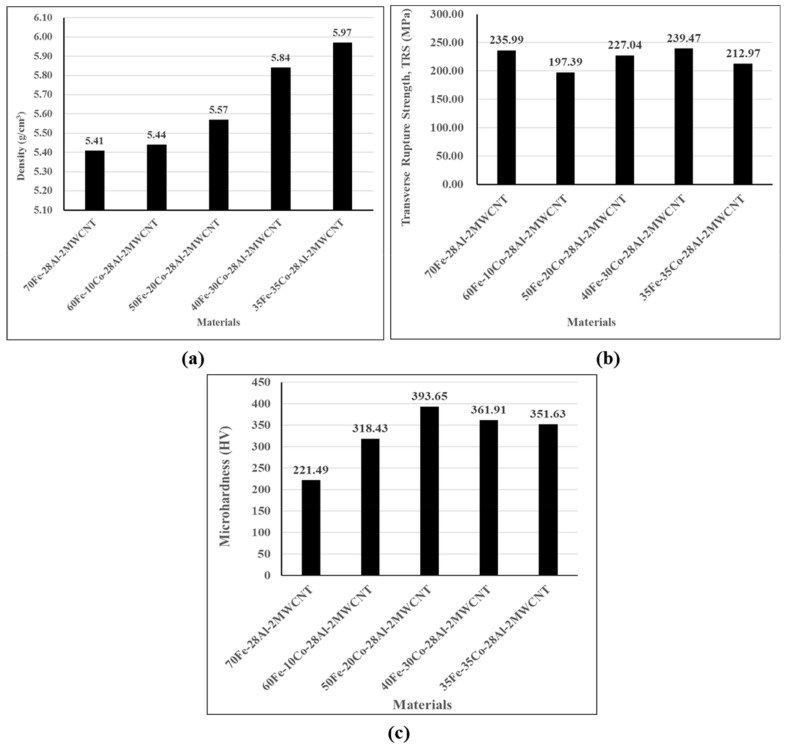
Effect of Co volume content on (**a**) volume density; (**b**) transverse rupture strength (TRS); and (**c**) Vickers microhardness of Fe-Co-Al-MWCNT nanocomposites.

The material densities were calculated as the ratio of the mass-to-volume of each nanocomposite, and the results are shown in Figure 12a. As expected, the density of composites increased with the Co volume content and their values were between 5.41 g/cm3 and 5.97 g/cm3. This can be explained by the higher density of Co material than that of other materials present in composites.

Figure 12b summarizes the average transverse rupture strength (TRS). It can be seen that at first, the TRS has dropped when Co 10 vol% was used and then started to increase to the maximum for composite with Co 30 vol%, and then dropped again. In this work, the Fe-Co-based composites had generally lower TRS than the composite without Co nanoparticles except for 40Fe-30Co-28Al-2MWCNT, which had the TRS of 239.47 MPa against 235.99 MPa of 70Fe-28Al-2MWCNT. The composite flexural strengths (TRSs) were in the range of 197 MPa to 240 MPa.

The effect of Co content on microhardness showed that Co had improved the hardness of the composites, as shown in Figure 12c. The hardness was found to increase from HV 221.49 at Co 0 vol% to HV 393.65 at Co 20 vol% and then reduced to HV 351.63 at 35 vol%. Therefore, an increase of 43.73% in Vickers hardness was achieved by incorporating Co 20 vol% in the composite. The enhancement of composite hardness due to cobalt incorporation is evidently due to the higher hardness of cobalt than iron [24]. Moreover, the variation in microhardness of the composites with respect to Co content may be explained by the change in microstructure, dislocation, interstitial defects and particle arrangements as a result of the manufacturing process.

## 4. Conclusions

In this work, the Fe-Al and Fe-Co-Al-based hybrid nanocomposites with MWCNT as reinforcements were fabricated by ball milling, followed by spark plasma sintering. The characterization of the composite morphology and magnetic, mechanical, and electrical properties was conducted according to the metallic particle combination. The morphology of composites distinctively showed the presence of all materials and their proper distribution. All composites showed a ferromagnetic behavior, and the magnetic properties were found to improve with an increase in iron content. The saturation magnetization continually increased to 167.22 A·m^2^/kg when the Fe content increased up to Fe 70 vol%. Although the incorporation of Co in Fe-Co-Al-MWCNT slightly reduced the saturation magnetization, it remarkably reduced the coercivity and retentivity to 42.68% and 47.4%, respectively. This suggests a significant improvement in the soft magnetic properties and reduction in hysteresis losses of the magnetic composite materials due to the use of Co nanoparticles. The manufactured composites in this study can be added to the group of semi-hard magnetic materials because their coercivities fall in the range of 1000 A/m and 100,000 A/m. Although the composites were magnetically harder than the conventional Sendust core, it was found that the composites with more than 60 vol% of ferromagnetic particles had better saturation magnetization than the Sendust core.

In general, the composites had good mechanical strength with values ranging between 173 MPa and 236 MPa for Fe-Al-MWCNT composites. The Co showed an enhancement of 1.25% in transverse rupture strength. The Fe content was found to be the leading element in hardness for the Fe-Al-based composites, where its increase led to an improvement of 57.14% in Vickers microhardness with the largest value of HV 221.49 which was further enhanced to HV 393.65 by incorporating 20 vol% of Co nanoparticles. We believe that the fabricated nanocomposites can be used as magnetic cores for electromagnetic devices.

## Figures and Tables

**Table 1:** Element volume ratio in Fe-Al-MWCNT hybrid nanocomposite materials. MWCNT: multi-walled carbon nanotube.

Composites	Materials ID	Fe (vol%)	Al (vol%)	MWCNT (vol%)
33Fe-65Al-2MWCNT	M1	33	65	2
38Fe-60Al-2MWCNT	M2	38	60	2
43Fe-55Al-2MWCNT	M3	43	55	2
48Fe-50Al-2MWCNT	M4	48	50	2
50Fe-48Al-2MWCNT	M5	50	48	2
55Fe-43Al-2MWCNT	M6	55	43	2
60Fe-38Al-2MWCNT	M7	60	38	2
65Fe-33Al-2MWCNT	M8	65	33	2
70Fe-28Al-2MWCNT	M9	70	28	2

**Table 2:** Element volume ratio in Fe-Co-Al-MWCNT hybrid nanocomposite materials.

Composites	ID	Fe	Co	Al	MWCNT
		(vol%)	(vol%)	(vol%)	(vol%)
70Fe-28Al-2MWCNT	M9	70	0	28	2
60Fe-10Co-28Al-2MWCNT	M10	60	10	28	2
50Fe-20Co-28Al-2MWCNT	M11	50	20	28	2
40Fe-30Co-28Al-2MWCNT	M12	40	30	28	2
35Fe-35Co-28Al-2MWCNT	M13	35	35	28	2
